# How the Dimensions of Plant Filler Particles Affect the Oxidation-Resistant Characteristics of Polyethylene-Based Composite Materials

**DOI:** 10.3390/ma17194825

**Published:** 2024-09-30

**Authors:** Joanna Aniśko, Paulina Kosmela, Joanna Cichocka, Jacek Andrzejewski, Mateusz Barczewski

**Affiliations:** 1 Institute of Materials Technology, Poznan University of Technology, Piotrowo 3, 61-138 Poznan, Poland; jacek.andrzejewski@put.poznan.pl (J.A.); mateusz.barczewski@put.poznan.pl (M.B.); 2 Department of Polymer Technology, Gdansk University of Technology, Narutowicza 11/12, 80-233 Gdansk, Poland; paulina.kosmela@pg.edu.pl; 3 Center for Advanced Technologies, Adam Mickiewicz University, Uniwersytetu Poznańskiego 10, 61-614 Poznan, Poland; joanna.cichocka@amu.edu.pl

**Keywords:** polyethylene, antioxidant, composite, natural composites, plant filler, waste valorization, black tea, tea, active filler, self-stabilizing composites

## Abstract

This study analyzed the possibility of using plant-originated waste materials (black and green tea dust) as functional polyethylene fillers. The dependence between the size of the filler particles and their antioxidant potential is discussed. Six fractions were selected: below 50 µm, 50–100 µm, 100–200 µm, 200–400 µm, 400–630 µm and 630–800 µm. Significant differences between the effect of particle size and the antioxidant properties of black and green tea were found using the extraction method to analyze antioxidant activity (DPPH method) and total phenolic content (Folin-Ciocalteu method), suggesting a higher potential for using green tea as a filler with antioxidant properties, as well as the benefits of finer active filler distribution. Biomass waste fillers were mixed with low-density polyethylene LDPE SEB 853 I’m Green^®^, Braskem. Those samples were oxidized at 100 °C for 5 and 15 days to investigate the radical scavenging properties of fillers in composites. Fourier transform infrared spectroscopic studies show that the addition of both types of filler prevents the thermo-oxidation of polyethylene for 5 days. After 15 days, all samples except the BTW 400–630 and 630–800 µm exhibited oxidation. The mechanical properties of the LDPE and its’ composites were tested, and we noted an increased brittleness of neat LDPE after thermal oxidation. The addition of black tea particles above 100 µm in size prevents this behavior.

## 1. Introduction

The antioxidant properties of plant-origin materials are well-known and have been thoroughly investigated. Up-to-date research has shown that plant extracts exhibit comparable effectiveness to commercial petrochemical additives in polymer stabilization [1]. From the point of view of waste valorization, limiting biomass pre-processing steps to obtain filler with specific features from plant residues is crucial. It led to the emergence of interest and studies on using low-processed plant fragments rich in active compounds [2,3]. The application of wastes from the food industry rich in antioxidants as active fillers revealed their ability to stabilize polymers, which became a particular interest [4]. Several researchers have shown that grape waste from winemaking, turmeric waste, orange peel, and coffee grounds can be used as stabilizers for low-density polyethylene (LDPE), comparable to commercially available synthetic additives such as Irganox I1010 [4]. Other reasonable substitutions for synthetic phosphites (various Irganox grades) are pomace extracts and tomato oleoresin [5]. The chemical composition of the commercial antioxidants Irganox 1010 and 1076 is based on high molecular weight phenolic compounds, whereas butylated hydroxytoluene (BHT), a low molecular phenolic antioxidant [6], as it was reported, is a potential liver carcinogen for rats [7]. The migration of BHT from thin high-density polyethylene (HDPE) plates to liquid and solid foods such as corn oil, mayonnaise, and orange juice has been confirmed by Till et al. [8]. This effect highlights why it is essential to investigate further the possibilities of replacing these harmful compounds with naturally available antioxidants.

The black and green tea antioxidant properties has been known for a long time [9]. Black tea contains 36% phenolic compounds, the dominant compound group in its composition [10]. It is prepared by oxidizing green tea and has different phenolic compounds: thearubigins and theaflavins [11]. These two beverages are at the top of global tea consumption, with 78% and 20% of the total share on the market for black and green tea, respectively [12]. The main antioxidant components in black tea are theaflavins, thearubigins, and catechins, which do not convert from green tea catechins to black tea polyphenols [13]. In the composition of green tea, the catechins alone account for 25 to 35% of the dry weight; the six main derivatives of these flavonoids are (+)-catechin, (−)-epicatechin, (+)-gallocatechin, (−)-epicatechin gallate, (−)-epigallocatechin, and (−)-epigallocatechin gallate [14]. Due to the formation of black tea from fresh green tea leaves through oxidation and polymerization, the amount of polyphenols decreases, and therefore, the antioxidant activity decreases [9,13]. According to Zhao et al. [15], epicatechin and epicatechin gallate are the only distinct flavonoids in black tea’s composition, whereas, for nine different spices of green tea, all of the flavonoids previously mentioned above were present. The various chemical compositions of these two teas result in higher antioxidant activity in green tea [16]. Aside from the superior antioxidant properties of green tea, the antioxidant activity of catechins extracted from green tea (epigallocatechin EGC, epicatechin gallate ECG, epigallocatechin gallate EGCG) and black tea (theaflavin, theaflavin monogallate, theaflavin digallate) exhibit higher inhibition in the DPPH test than the previously mentioned BHT [17]. Considering the high potential resulting from their chemical composition and high amounts of extractives, they may be proposed as efficient replacements for artificial stabilizers.

Green and black tea wastes have been used in polymer composites. Several works discuss the introduction of green tea extract into food packaging to decrease radical scavenging and increase the freshness of food products [18,19]. In the literature, there are works considering the usage of tea extracts or directly antioxidant compounds originating from teas. In the work performed by Shah et al., the flavonoid epigallocatechin gallate found in teas was used in ultrahigh molecular weight polyethylene to improve the stability of oxidation and microbial inhibition [20]. Very fine particles (<35 µm) of brewed tea were used to obtain composite films based on cellulose with biodegradable properties [21]. Some works on the utilization of tea wastes, such as waste from the Lipton tea mill, were used in high concentrations (up to 50 wt%) in HDPE and polypropylene (PP) based composites [22].

This study investigates the stabilization possibilities of low-density polyethylene fillers and the influence of the filler size on their functional properties. The filler size, shape, and distribution highly influence the mechanical properties of thermoplastic composites. Reduced filler size allows effective reinforcing effects, increasing the tensile strength and modulus of composites [23]. The filler particle size also influences the extraction efficiency; the smaller the particle, the shorter the extraction time (Qu et al., 2010) [21]. However, this applies to extraction in an aqueous environment in the current state of knowledge. The migration of low-molecular-weight compounds from the low-processed biomass used as a filler to the polymer is an effect still unexplored and not described qualitatively and quantitatively. The understanding of the effectiveness of the interaction of active compounds is so far based on the description of the final properties of the composite subjected to spectroscopic evaluation or thermal analysis [24,25,26,27]. The research proposed in this study fills the gap in the state of knowledge with information including the degree of filler fragmentation. Filler fragmentation results in an increased interphase surface in the composite between the polymer and the dispersed filler particles, which affects the beneficial interaction of migrating antioxidants. Considering the industrially oriented research and the interest in the implementation aspects, the ranges of favorable fractionation of the filler selected, thanks to this work, will allow researchers to obtain data that will limit the cost and time-consuming processes of biomass pre-processing. The research conducted in this paper investigates the influence of particle size on both the mechanical and antioxidant properties of tea waste on the polyethylene matrix.

## 2. Experimental

### 2.1. Materials and Sample Preparation

To prepare samples for further investigation, black and green tea wastes collected as dust formed during the packing of tea into teabags were separated into six fractions: <50 µm, 50–100 µm, 100–200 µm, 200–400 µm, 400–630 µm, and 630–800 µm. To do this, the vibratory sieve analyzer (Fritsch Analysette 3 PRO, Idar-Oberstein, Germany) was used. This material is classified as waste in the production system. The matrix used in this study is the bio-based low-density polyethylene LDPE SEB 853 I’m Green^®^ obtained from Braskem (São Paulo, Brazil).

**Figure 1:**
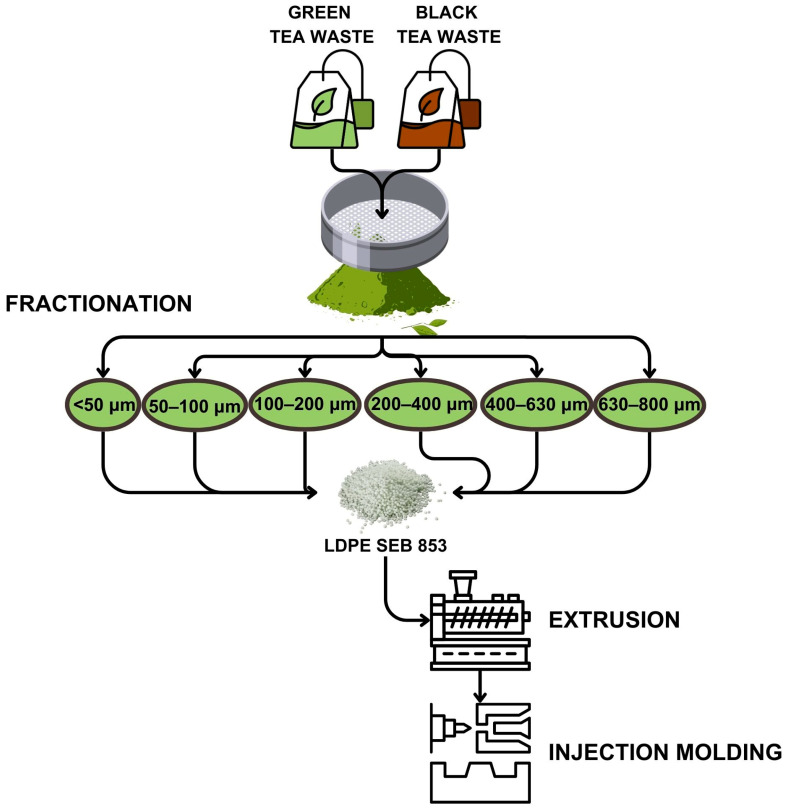
Scheme of sample preparation for testing.

Selected fillers were introduced into the LDPE matrix in the amount of 1 wt%. The selected amount of active filler is based on our previous work, in which this low content of natural fillers provided increases in oxidation induction time compared to neat LDPE [2,28,29,30]. This selection will also draw more attention to filler particle size rather than its volume in the polymer matrix. Composites with these different size fillers were prepared on a ZAMAK EH16.2D (Skawina, Poland) co-rotating twin screw extruder operating at 150 rpm. The temperature profile from the feed hopper to the nozzle was 90–110–120–130–140 °C. The final samples in the form of 100 × 100 × 4 mm and 100 × 100 × 2 mm plates were obtained by injection molding using an Engel e-mac 170/50 (Schwertberg, Austria) with the highest processing temperature of 140 °C. Other employed parameters are the injection pressure, which was 1200 bar, a holding pressure of 600 bar, and holding and cooling times of 10 and 20 s, respectively. A flow chart showing the composite manufacturing procedure is presented in Figure 1.

### 2.2. Thermal Oxidation

The composite samples were subjected to thermal oxidation in an air-circulating oven at 100 °C. They were removed after 5 and 15 days to evaluate their properties after degradation.

### 2.3. Methods

To investigate the antioxidant properties of green tea and black tea waste, the DPPH free radical scavenging assay was performed using the 2,2-diphenyl-1-picrylhydrazyl radical. The methanol solution of DPPH is characterized by a characteristic violet color, which fades after an antioxidant addition. This effect can be captured and measured with a Schott UviLine 9400 (Mainz, Germany) UV-Vis spectrophotometer, operating in the range of 190–1100 nm with a resolution of 1 nm. To calculate the antioxidant activity of the black and green tea waste, the extracts were prepared with 50 mg of dried material and 50 mL of methanol using a magnetic stirrer (25 °C, 300 rpm, 30 min). The resulting extracts were filtered using round filters with medium-speed filtration. This assay was performed using a 63 µM solution of DPPH in methanol [31]. In dark flasks, DPPH solution was added to 0.15 mL of filler extract to replenish up to 3 mL. The flasks were kept closed and stored in the dark for 30 min before measurement, which was performed using a UV-Vis spectrophotometer at 517 nm with quartz cuvettes. All measured inhibition values (1) were also expressed as Trolox equivalents. Three repetitions for each series were undertaken.

(1) $$AA=\frac{A_{control}-A_{sample}}{A_{control}}$$

where, *A_control_*—absorbance of DPPH solution at 517 nm and *A_sample_*—absorbance of the sample at 517 nm.

The Folin-Ciocalteu method was used to assess the quantification of total phenolic compounds. This analysis was performed following the protocol of Rebollo-Hernanz et al. [32], with suitable adaptations to the volume of the reagents. The same preparation procedure used to prepare the extracts was applied as in the antioxidant activity assay. In Falcon-type vials, 150 µL of methanol extract (1 g/L) was added and diluted with 2100 µL of distilled water, then 150 µL of Folin-Ciocalteu reagent was added and the mixture was left to rest for 3 min. After this time, the 20% aqueous solution of Na_2_CO_3_ was poured into each vial, and the mixture was well homogenized using a vortex stirrer and left to rest for 2 h. As before, the colorimetric reaction was quantitively assessed using a Schott UviLine 9400 UV-Vis spectrophotometer, and absorbance measurements were taken at 750 nm. The absorbances obtained for each sample (in triplicate) were expressed as gallic acid equivalents.

The chemical composition of the black and green tea waste fillers was evaluated using Fourier transform infrared spectroscopy in ATR mode. The parameters for this method were: resolution 4 cm^−1^, wavenumber range 4000–400 cm^−1^, and 64 scans. The apparatus used for this evaluation was a Jasco FT/IR-4600 spectrometer (Tokyo, Japan). The protocol followed in this test includes noise elimination, CO_2_ reduction, H_2_O reduction, ATR correction, auto baseline correction, and smoothing, in that exact order.

The surface areas of the analyzed fillers and their nitrogen adsorption isotherms were determined using an accelerated surface area and porosimetry apparatus (Micromeritics ASAP 2420 (Norcross, GA, USA)) following the Brunauer–Emmett–Teller (BET) method at 77 K. All materials were degassed at 120 °C for 12 h in a vacuum chamber before measurement. The multipoint BET method used adsorption data under relative pressure (p/p0) to determine the specific surface area.

Microscopic images of the composite samples were taken using an Opta-Tech MB200s optical microscope (Warsaw, Poland). The microscope was connected to a Meiji Techno HD2600T camera (Tokyo, Japan). The images were observed at 40× magnification.

Thermogravimetric analysis was performed to evaluate the thermal stability of neat polyethylene, its composites, and plant-based fillers. Six fractions of black and green tea waste were analyzed using thermogravimetry. The apparatus used in this study was the Netzsch TG 209 F1 Libra apparatus (Selb, Germany). The 10 mg samples were heated in Al_2_O_3_ crucibles from 25 °C to 900 °C at 10 °C/min in a nitrogen flow 20 mL/min. The thermogravimetric parameters extracted from this test were T_95_, T_90_, and T_50_, which are temperatures at 5, 10 and 50% of mass loss, respectively, the DTG peak (temperature at highest mass loss rate), and residual mass.

The ash content of the biomass fillers was investigated during combustion at 900 °C in an air atmosphere. The analysis was performed using a muffle furnace and ceramic crucibles. The ash content was defined as the residue from the incineration. The batch weight of each sample was about 2 g.

To analyze the potential stabilizing effect of added antioxidant fillers, the oxidation induction time (OIT) test was performed. This is one of the differential scanning calorimetry (DSC) techniques in which 5 mg samples are placed in aluminum crucibles equipped with pierced lids in an oxygen atmosphere. The temperature program consisted of heating from 20 to 190 °C at the rate of 20 °C/min and performing the isothermal step at 190 °C for 5 min in a nitrogen atmosphere. After that, the gas atmosphere was switched to oxygen, and the measurement continued until oxidation occurred. Measurements were made using a Netzsch 204 F1 Phoenix instrument (Selb, Germany).

To investigate the chemical changes in low-density polyethylene composites, Fourier transform infrared spectroscopy was used before and after thermal oxidation. The apparatus used in this was a Jasco FT/IR-4600 Fourier transform infrared spectrometer (Tokyo, Japan). Measurements were made by taking 64 scans at a resolution of 4 cm^−1^ in the wavenumber range of 4000–400 cm^−1^. The data obtained for the thermally oxidized samples were used to calculate the carbonyl index (*CI*) using Equation (2). This index defines the rate of oxidation of polyethylene.

(2) $$CI=\left(\frac{A_{\left(C=O\right)}}{A_{0}}\right)$$

where *A_C_*_=*O*_ is the absorbance of the carbonyl group (1800–1650 cm^−1^) and *A*_0_ is the absorbance of methylene (CH_2_) from 1500 to 1420 cm^−1^, which was selected as a reference because it shows minimal affection for thermal oxidation [33,34].

The static tensile test was performed using a universal Zwick Roel Z010 test machine to evaluate the samples’ Young moduli and tensile strengths before and after thermal oxidation. The test was carried out according to the PN-EN ISO 527 [35] standard with a crosshead speed of 1 mm/min during the Young’s modulus determination (up to an elongation of 0.2%) and 100 mm/min for the rest. Specimens with dimensions of 10 mm in width, 100 mm in length, and 2 mm in thickness were tested. The average values of five samples were analyzed.

The impact strength of composites was determined using the Charpy impact test. The test was conducted using the Zwick/Roel Hit 25 testing machine, according to the PN-EN ISO 179 [36] standard, with samples measuring 80 mm long, 10 mm wide, and 4 mm thick. In this study, at least five notched-shaped samples were examined and broken with a 5 J hammer.

The samples used for the contact angle analysis were made out of injection-molded materials. For the contact angle analysis, an Ossila contact angle goniometer (Sheffield, UK) equipped with a 25 µL glass syringe to dispense droplets of distilled water was used. To measure the angle formed between the drop and sample, we used the Ossila Contact Angle v 3.0.9.1 software. Contact angle data are presented as an average angle based on at least three measurements.

## 3. Results and Discussion

### 3.1. Characterization of Fillers

**Figure 2:**
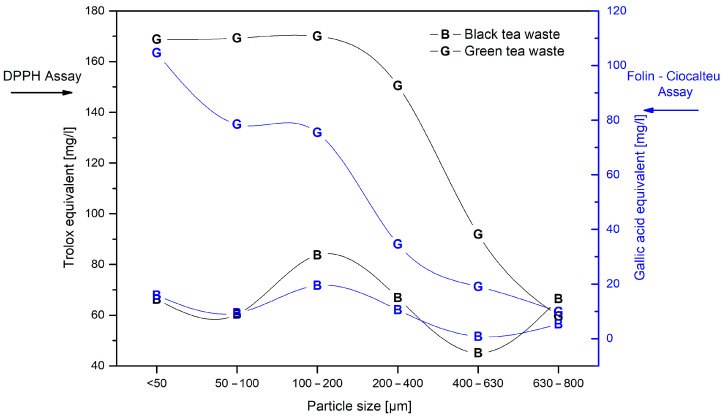
Antioxidant properties as a function of filler particle size.

The DPPH assay was used to characterize fillers’ antioxidant properties; antioxidant activity was calculated according to the Trolox equivalent (Figure 2). Crucial differences were observed between black and green tea extracts. The extracts obtained from green tea waste had higher antioxidant activity than black tea waste extracts. Similar values were determined for extracts made from fillers with particle sizes between 630 and 800 µm for both materials. The minimized effect of oxidation on green tea leaves causes the highest antioxidant properties; thus, the fermentation of black tea leaves makes this a weaker free radical scavenger. The chemical explanation for this lies in the significant amount of catechins in green tea [15,37]. Due to non-fermentation, green tea has a higher catechin content, which is oxidized during fermentation to black tea, and new compounds are formed, such as theaflavins and thearubigins [38]. The purpose of this work was to investigate whether the antioxidant filler size has an impact on the stabilization effect. The first step was to investigate the antioxidant properties of methanolic extracts made out of tea waste of different particle sizes. This characteristic has the highest impact on green tea extracts, and the highest antioxidant activity is found in extracts made using the smallest filler particles. This correlation is not so clear when it comes to black tea waste extract. The highest antioxidant activity is found in series with particle sizes within the 100 to 200 µm range. The total phenolic content expressed as gallic acid equivalent have the similar changes as the DPPH assay results for the same kind of material. The dependence between material particle size and antioxidant properties is more visible for green tea waste extract, but it is worth noting that this affects samples below 400 µm. This difference fades away above this filler size. The results presented align with the literature, and different studies have proved that a smaller particle size provides the highest extraction efficiency [39,40]. The smaller the particle is, the shorter the mass transfer distance and the larger the surface area; this results in increased extraction efficiency and reduced extraction time [24].

**Figure 3:**
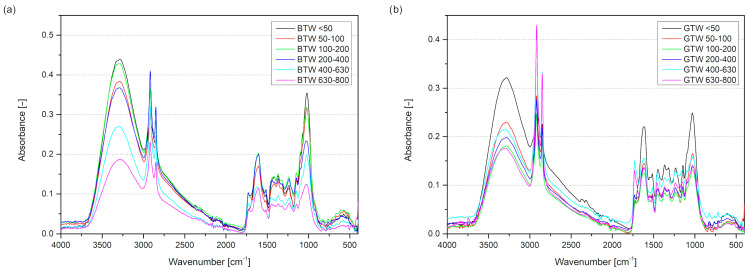
The FTIR spectra of black tea waste (**a**) and green tea waste samples (**b**).

**Figure 4:**
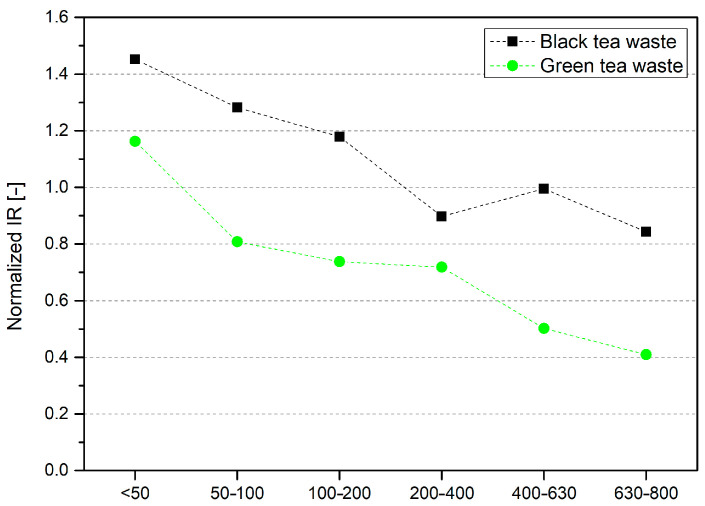
Normalized IR absorption for O–H bond.

Fourier transform infrared spectroscopy was used to investigate the chemical composition of plant-based fillers (Figure 3). Samples were pressed using a hydraulic press to pellets with a diameter of 15 mm in order to condense powders and eliminate air interference. The obtained spectra for biomass samples have several characteristic peaks. Each identified peak’s position and description are shown in Table 1. Mainly, all of the detected peaks were similar in both materials. The only difference appeared between 1540 and 1650 cm^−1^, where for green tea waste samples, the peaks associated with the N–H bond were detected [41]. Two identified bands at 1548 cm^−1^ and 1621 cm^−1^ were associated with N–H stretching vibrations. The chemical composition of tea leaves not only includes polyphenolic compounds and lignocellulosic structure but also the alkaloids like theobromine and caffeine with N–H bonds in their structures [13]. Green tea leaves contain almost twice as many total purine alkaloids as black tea, which is why these peaks appear only in green tea wastes [42]. Comparisons of samples with different particle sizes indicate an apparent reduction in the intensity of the O–H bond peak. To visualize the changes in this peak’s intensity, the IR absorption was normalized using the absorbance of the C–H peak at 2917 cm^−1^ (Figure 4). The amount of –OH groups in polyphenolic compounds indicates their antioxidant potential; the higher, the better [43]. With the particles’ increasing size, the normalized O–H peak absorption decreases gradually, and lower values are observed for green tea waste samples. The dominant group of antioxidant compounds found in tea leaves are flavonoids, mainly catechins. The presence of the pyrogallol group in its structure or galloylation of the 3-hydroxyl group increases its antioxidant activity; the structures of this compound are aromatic rings with three hydroxyl groups [43,44].

**Figure 5:**
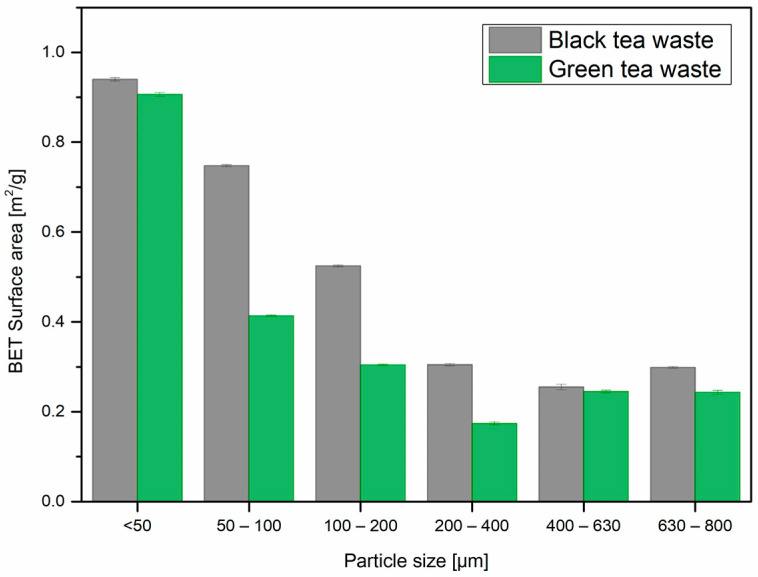
Results of BET surface area of the fillers measurements in relation to their particle size.

Dependencies between particle size and surface area were also investigated. The antioxidant activity is higher for smaller particles and, thus, for particles with a larger specific surface area. Figure 5 presents surface areas as a function of filler particle size for both filler types; the smaller the particles, the higher the BET surface area. Zhong and co-workers reported a similar tendency for the pomegranate peel, which was ground using a bed jet mill, employing high-impact particle collision [47]. In this case, tea waste comes from the industry as a by-product of the transformation of crushed leaves, which causes more intense defragmentation of the particles and eventually rejects them from further production steps. The mentioned study not only confirms the dependency between filler size and surface area observed by us, but also measured the antioxidant properties of different fractions of pomegranate peel powders. The extraction of phenols and flavone compounds is enhanced for smaller particles, resulting in more significant antioxidant activity.

**Figure 6:**
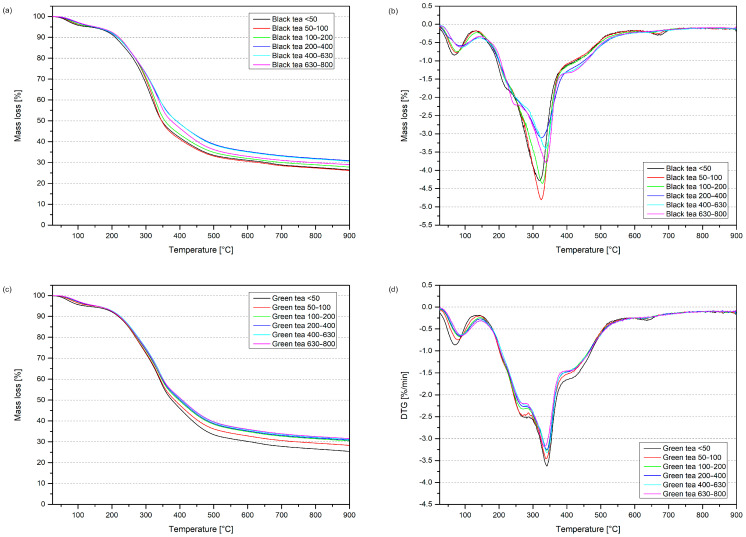
Thermogravimetric analysis of waste fillers: black tea (**a**,**b**); green tea (**c**,**d**).

The thermal stability of the fillers was determined by thermogravimetry (TGA), which results in curves of mass loss and the first derivative of mass (DTG) vs. temperature (Figure 6). Additional data, including the temperature corresponding to mass loss at 5, 10, and 50%, residual mass, and information about the characteristic values of peaks recorded in the DTG curve, are presented in Table 2. After the thermogravimetric test, the difference in degradation temperatures between the tested fillers was noted. Differences were visible not only between black and green tea but also between fractions. In Figure 4, the thermograms of natural fillers are visualized. At first sight, the differences in curves are noticeable, as are the overlapping degradation effects. Deconvolution was performed using OriginPro 8 software with a Gaussian fit to distinguish these peaks. Six separate peaks were distinguished, and each of them can be assigned to a specific degradation process. The first peak is related to the evaporation of water from plant-based materials. The following three peaks, which overlap each other, are characteristic of the degradation of hemicellulose, cellulose, and lignin, respectively; their recorded temperatures align with the literature data [48]. The fifth peak is only observed for the smallest fractions of black tea but appears in almost every sample of green tea (except for the largest size). This visible mass loss peak is established as a carbonization reaction and the formation of ashes, in which the heaviest compounds evaporate [49]. It is also worth noticing that the share in the overlapped peak of each substituent differs between black tea and green tea. This observation may be correlated with the lignocellulosic composition of each tea; the cellulose content is at a comparable level, but the hemicellulose and lignin contents are higher for black tea waste [50]. The higher amounts can explain the more intense thermal response and overlapping peaks. Black tea waste fractions have an increasing peak temperature, with the highest degradation rate due to increasing particle size, but it is still lower than the DTG peak temperature for green tea. The residual mass from the thermogravimetric study is approximately 30% after pyrolysis in a nitrogen atmosphere. After combustion in the muffle furnace, the ash content decreased to approximately 5%, with the exceptions of BTW 50 and BTW 50–100, in which the inorganic residue after combustion is higher, 9.1% and 7.7%, respectively.

### 3.2. Characterization of Composite

**Figure 7:**
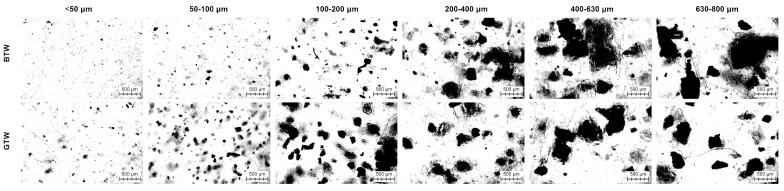
Microscopic images of composite samples.

Microscopic images of the composite samples are shown in Figure 7 to demonstrate the distribution of herbal fillers. The top row represents samples filled with black tea waste. Compared to green tea waste samples, we can observe a very gradual increase in particle size. Composite samples filled with green tea powder have particle sizes closer to the upper limit of the fraction, up to 200 µm, and above that, the particles are significantly lower than for black tea samples. The tea powder is formed during the grinding of the tea leaves; the difference between the particle sizes can be related to the different brittleness of those leaves. The distribution of both of these fillers can be considered even. Particles did not form large clusters and agglomerations.

The oxidation induction time is a thermal parameter commonly used to assess the resistance to oxidation in the molten state of polymers and, therefore, is often used as a basis for determining the stabilization efficiency of various additives. Adding fillers rich in compounds with antioxidant properties that can migrate into a polymeric matrix extends the oxygen induction time compared to neat polyethylene. The results of the OIT evaluation for polyethylene and its composites are presented collectively in Table 3. Generally, the higher OIT value is for samples using green tea waste. In comparisons between samples with different particle sizes, there is a limit above which OIT significantly decreases. That limit is 200 µm. The limitation of the stabilization effect of black and green tea used as a filler above this particle size can be caused by the lower distribution density of particles with more significant dimensions. Samples taken for this test were approximately 5 mg each. Due to this, the probe sample could not represent the ideal whole volume, in which fillers were added as a 1 wt%. In studies performed, adding 1 wt% of antioxidant active filler with larger particles causes even a lower OIT than the neat matrix [2] or no significant increase [30]. The additional explanation is based on a higher release of antioxidant compounds into the methanolic extract; smaller particles have better efficiency if this behavior is directly transferred into the polyethylene composites. Smaller particles with higher surface areas may cause an increased migration of low molecular compounds into the polymer matrix, thereby increasing the OIT value, based on data for the extractivities of these compounds in solvents [51].

Table 4 summarizes the thermal properties of polyethylene and its composites containing BTW and GTW. Thermogravimetric evaluation of plant-based composites did not show any significant differences in the temperature of the beginning of degradation, considered in this case as the temperature at 5% mass loss T_95%_. The same applies to the temperature at 10% and 50% mass loss, as well as the DTG peak temperature. The addition of just 1 wt% of black and green tea filler did not influence the thermal decomposition of low-density polyethylene. The processing conditions for a polymer and its composite can be fixed similarly without the risk of overheating and degradation of the plant-based filler.

**Figure 8:**
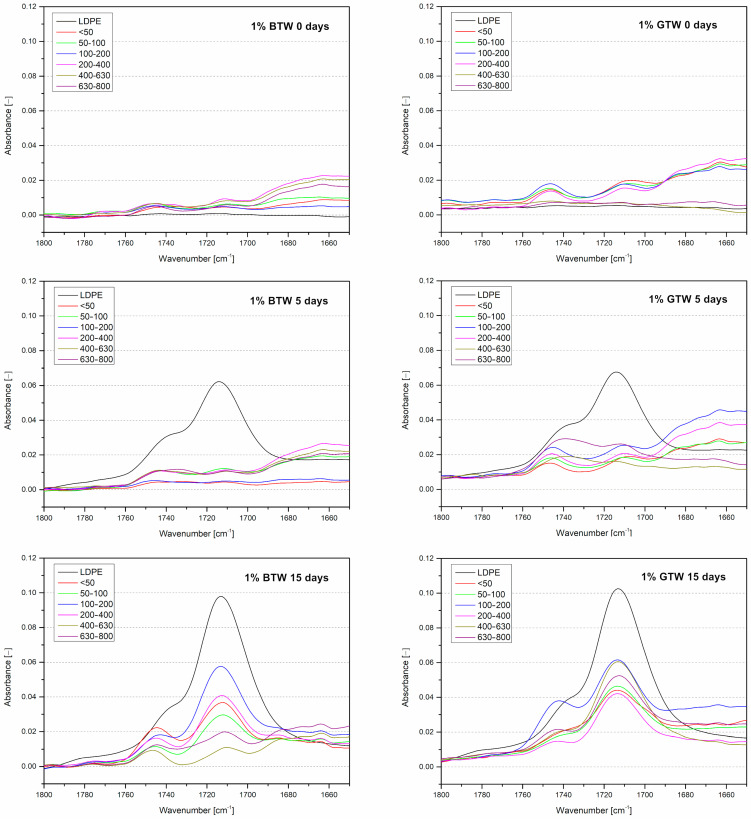
Materials’ spectra before and after thermal aging after 5 and 15 days ranged from 1650 to 1800 cm^−1^.

The next part of this study consists of an analysis of thermally oxidized samples as a supplement to the OIT analysis. The analysis is provided to prepare a multi-criteria indirect assessment of the effectiveness of the impact of fillers of different fractions and origins on the polymer matrix. The first test conducted on the thermally aged samples was Fourier transform infrared spectroscopy (FTIR). This is the most helpful tool for investigating changes in chemical composition, especially in the case of polyethylene analysis for quantifying the carbonyl group content that forms as an effect of oxidation [52]. In Figure 8, the spectra of materials before and after thermal aging in the range of the wavenumber where the carbonyl peak can occur are presented. After five days of exposure to elevated temperatures, the carbonyl peak did not appear in the composite samples. Prolonging the thermal oxidation resulted in the appearance of a band characteristic of carbonyl formation in composite materials.

**Figure 9:**
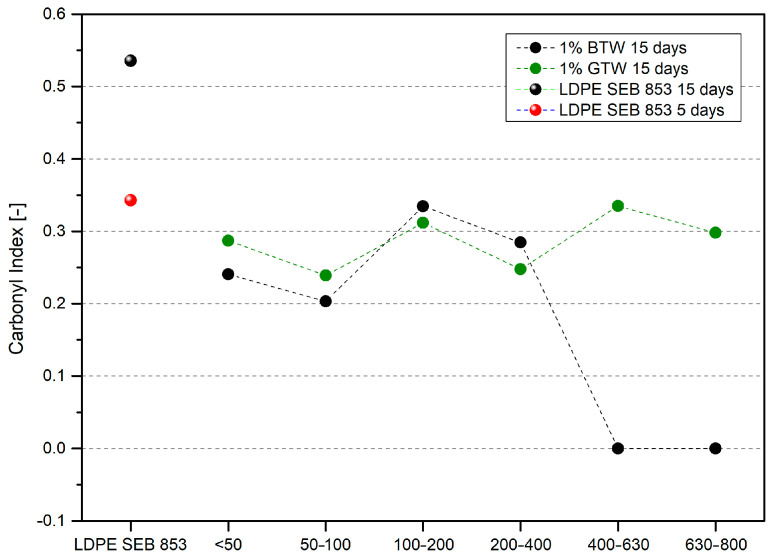
Changes in the carbonyl index for samples after thermal oxidation.

The carbonyl index was calculated according to Equation (2) to better investigate the relation of oxidation and the particle sizes of filler. Figure 9 presents carbonyl indexes for LDPE after 5 and 15 days of thermal oxidation and after 15 days for composites. The astonishing observed behavior is the unoxidized structure of the samples with black tea waste filler larger than 400 µm. Analysis of the antioxidant properties of the black and green tea extracts showed that green tea is richer in antioxidant compounds. However, accelerated aging tests under oxidizing conditions showed that black tea reveals better scavenging of free radicals formed during polyethylene oxidation [53]. Not only was the unexpected behavior of black tea observed, but also the least favorable particle fractions from the DPPH analytical point of view (400–800 μm) protected the material from oxidation. The processing parameters introduced provided a uniform distribution of both fillers in the polymer matrix (Figure 5), but larger particles ensure greater contact between the filler edges and the polymer, allowing the low-molecular-weight phytochemicals to migrate more easily from the natural fillers into the polymer matrix.

**Figure 10:**
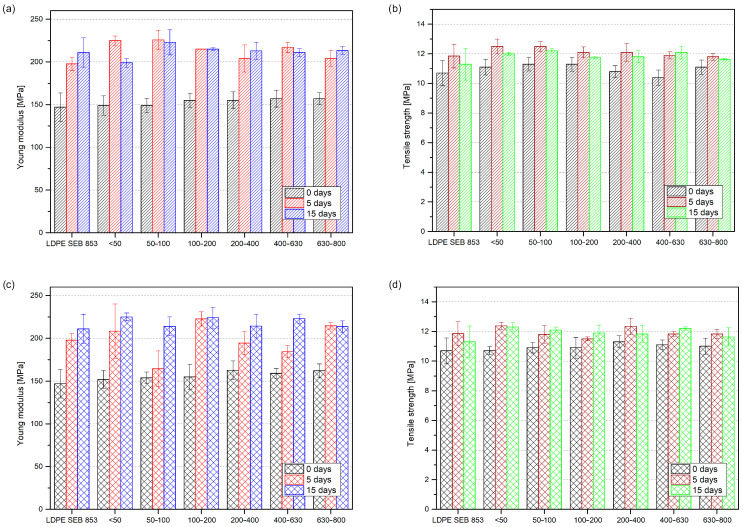
Mechanical properties obtained in static tensile test for samples of 1% BTW (**a**,**b**) and 1% GTW (**c**,**d**) samples before and after thermal oxidation.

Figure 10 summarizes the mechanical properties of LDPE and composites containing different BTW and GTW fractions obtained in the static tensile test before and after the thermal aging process. The Young’s modulus of non-degraded samples increased with an increase in particle size from 147 MPa for neat LDPE to 157 MPa for black tea waste of 630–800 µm and 162 MPa for green tea waste. The relationship between particle size and its value is not linear for tensile strength. The highest tensile strength was observed for 1% BTW, with 50–100 and 100–200 µm in turn; for 1% GTW, the 200–400 µm series showed the higher tensile strength. The addition of micrometric particles to a polymer matrix increased the stiffness of the composites, which is more prominent for smaller particles [54]. This effect is more pronounced in the case of composites filled with a higher amount of filler, and as the proportion of filler increases, the effect becomes more noticeable [23]. In the case of this study, only 1 wt% of particle fillers was added to investigate the influence of particle size on their stabilizing properties. This amount of filler did not have an additional high stiffening effect, and contrary to expectations, the largest particles caused the highest increase in Young’s modulus. The tensile strength of the composite samples did not increase or decrease significantly; the average value of all samples with BTW was 10.9 MPa with a standard deviation of 0.3 MPa, while for samples with green tea waste, it was 10.9 ± 0.2 MPa. After thermal oxidation, the modulus of the elasticity of the LDPE samples and their composites increased. The neat LDPE samples increased their Young’s modulus by 50.7 MPa after 5 days of oxidation and 64.0 MPa after 15 days. Thermal oxidation of polyethylene leads to chain-scission simultaneously with crosslinking, the first causing the formation of lower molecular weight molecules and the second forming molecules with higher molecular weights [55]. This effect is characteristic of LDPE, which increases its Young’s modulus during multiple reprocesses due to more chain entanglements between shorter polymer chains [55].

In the case of LDPE filled with black tea waste, the highest increase in stiffness is observed for samples containing particles below 100 µm thermo-oxidized for 5 days. In a series of samples oxidized for 15 days the same sample (50–100 µm) exhibited the highest increase. The rise of Young’s modulus for samples with green tea waste oxidized for 5 days is smaller than that of samples oxidized for 15 days. LDPE with 1% GTW in particle sizes below 50 µm exhibited the highest increase in its modulus of elasticity; the modulus rose by 73.0 MPa. Materials are compared in series to unaged samples. This increase in the elastic modulus leads to a slight increase in the tensile strength of oxidized samples, including green and black tea; this improvement is proportional to the increase in tensile strength. According to Mourad et al., the tensile strength of LDPE thermally aged at 100 °C also increases slightly with the elongation of the degradation time up to 14 days [56].

**Figure 11:**
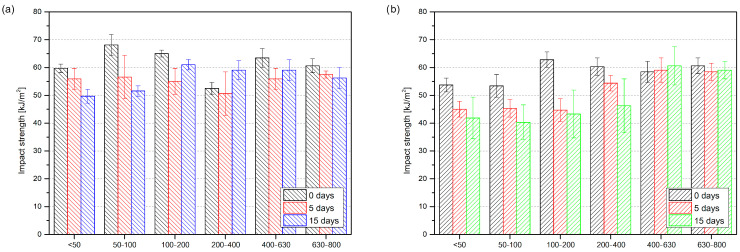
Impact strength of low-density polyethylene with black tea waste (**a**) and green tea waste (**b**) before and after thermal oxidation.

**Figure 12:**
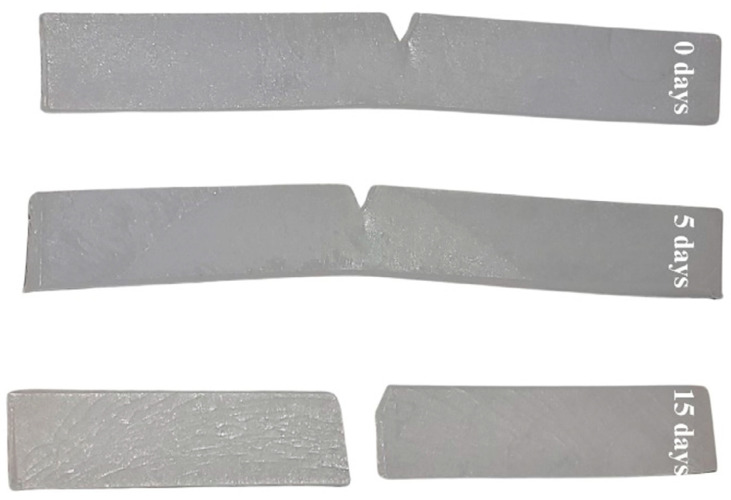
The photography of LDPE samples after impact strength test.

Thermally aged materials were also investigated in terms of their impact strength (Figure 11). The unmodified polyethylene, before oxidation, did not break during the impact strength test. Nor did the LDPE samples after 5 days of thermal aging. A different behavior was observed for LDPE after 15 days of oxidation; the samples became very brittle, and their impact strength was 1.1 ± 0.2 kJ/m^2^. Photographs of the LDPE samples after the impact strength test are presented in Figure 12. The effect of increasing brittleness and surface cracking on oxidized polyethylene has been described widely before [57]. The same testing procedure was applied to composite samples to investigate the influence of the introduced natural filler. All of the composites broke during the measurements. Using black tea waste as an efficient stabilizer for LDPE, fillers with particle sizes greater than 100 µm prevented the sudden decrease in impact strength reported after the thermal aging of LDPE. In the case of green tea waste, only the addition of filler particles of a size greater than 400 µm prevents increasing embrittlement. Again, the excellent antioxidant properties of green tea extracts did not make them into great natural stabilizers for polyethylene; black tea waste modifying efficiency slightly exceeds green tea waste in this context.

**Figure 13:**
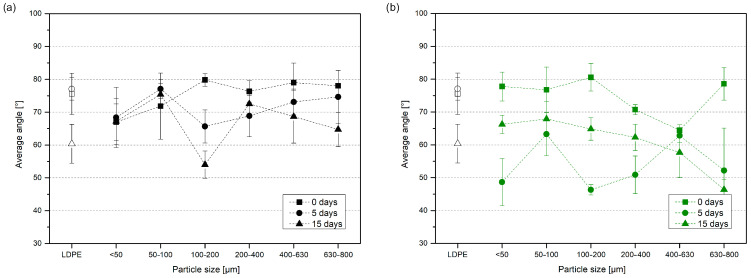
Contact angle of low-density polyethylene and its composites with black tea waste (**a**) and green tea waste (**b**) before and after thermal oxidation.

Composites filled with natural fillers may vary in surface contact angle since the polar polymer matrix is mixed with nonpolar, highly hydrophilic fillers due to not only the share of individual components but also processing resulting in different dispersion of the particles and creation of hydrophobic layer at the sample’s surface [26]. The contact angle test using distilled water was performed to assess the changes in hydrophilicity of composites containing fillers with different fractions. In Figure 13, the values of the contact angle for neat LDPE, its composites, and samples after thermal degradation are presented. The addition of natural waste fillers did not cause a significant decrease in contact angle compared to neat LDPE. The previously mentioned increase in brittleness and cracking of the surface led to a reduction in the contact angle of LDPE. The same was observed for composite samples, but the green tea samples revealed a more significant value decrease. The influence of particle filler size cannot be clearly defined. Nor can we answer the question of whether the larger or smaller particles have more substantial effects on the surface properties of LDPE composites. According to Julienne et al., UV-aged LDPE films’ contact angle decreases with prolonged exposure time, as proven via AFM microscopy, resulting from increased roughness and micro-cracks [58].

## 4. Conclusions

Research conducted in this work demonstrates the influence of filler particle size on the properties of composites. The waste fillers were applied to increase the thermal stability of polyethylene. In the case of studies on the antioxidant properties of black and green tea extracts, waste green tea exhibits higher antioxidant activity and content of phenolic compounds, and the smaller the filler particle used for extraction, the higher the activity. Black tea waste samples have an apparently lower content of phenolic compounds and antioxidant activity. The particle size in BTW has a less significant effect on these properties. In order to evaluate the impact of the chemical composition of waste fillers on antioxidant activity, Fourier transform infrared spectroscopy was performed, including normalized IR absorption of the O–H band analysis. The increasing size of filler particles caused a decrease in normalized IR absorption, but surprisingly, the black tea waste fillers exhibited higher values. The number of hydroxyl groups in the compound indicates its antioxidant potential. The smaller particles exhibit a higher surface area, which enhances their ability to extract more antioxidant compounds. Green tea wastes exhibit higher thermal degradation temperatures in nitrogen than black tea waste. Additionally, the BTW fractions have an increasing temperature value for the peak with the highest degradation rate due to increasing particle size, but it is still lower than DTG peak temperature for green tea.

The most common method used to investigate the stabilizing effect in oxidizing conditions is to measure the oxidation induction time (OIT), which has resulted in higher times for samples with lower particle sizes (below 200 µm) and slightly higher for green tea waste. The opposite relationship was observed in the case of polymer composites subjected to thermal oxidation. Analyzing the carbonyl index of oxidized samples, introducing black tea from the two largest fractions prevents oxidation in LDPE, resulting in materials without a carbonyl band. Throughout the rest of the performed tests, samples with black tea waste exhibited a lower impact of oxidation. Composites with 1 wt% of GTW became more brittle after thermal oxidation; they had lower impact strength than samples with BTW. Adding 1 wt% of biomass into LDPE caused increases in Young’s modulus of non-degraded samples with increasing particle sizes. The samples’ thermal oxidation phenomenon caused an additional increase in their stiffness. Moreover, after thermal aging, samples exhibited more hydrophilic surfaces and lower water contact angles. These effects were more pronounced for composites with green tea waste.

To summarize, the introduction of plant-based fillers did not influence the degradation temperatures of the composite samples as much as unmodified polyethylene did. The expected effect of lower particle size being a better stabilizing agent is negligible in composites, whereas bigger black and green tea particles prevent the negative impact of thermal oxidation. Surprisingly, black tea waste can be described as a more efficient active filler with stabilizing ability than green tea waste when tested against thermal oxidation at 90 °C.

## Figures and Tables

**Table 1:** The FTIR peaks for black and green tea wastes with their assignments [40,41,45,46].

	Wavenumber [cm^−1^]		Assignment
	BTW	GTW	
1.	1019	1031	C–O deformation in primary alcohol
2.	1146	1145	C–O–C vibration in cellulose and hemicellulose
3.	1238	1233	C–H stretching
4.	1324	1319	Primary or secondary O–H bending (in-plane) and phenol or tertiary alcohol (O–H bend)
5.	1369	1366	CH_3_ bending
6.	1415	1447	C–H band in-plane deforming and stretching
7.	1516	1516	Aromatic skeletal with the C=C band in the aromatic ring
8.	−	1548	N–H bending vibrations
9.	1606	−	Aromatic skeletal with the C=C band in the aromatic ring
10.	−	1621	N–H bending vibrations or C–O bending vibrations
11.	2850	2849	C–H stretch
12.	2917	2917	C–H stretch
13.	3286	3284	O-H stretch

**Table 2:** Thermal parameters of different black and green tea waste fractions determined by TGA.

Waste Fillers	T_95_ [°C]	T_90_ [°C]	T_50_ [°C]	DTG					Residual Mass [%]	Ash [%]
				Peak 1 [°C]	Peak 2 [°C]	Peak 3 [°C]	Peak 4 [°C]	Peak 5 [°C]		
BTW < 50	133.0	209.7	346.6	71.3	252.2	316.9	407.2	661.1	26.5	9.1
BTW 50–100	149.7	217.0	346.3	75.5	279.8	323.7	426.9	643.8	26.2	7.7
BTW 100–200	145.6	217.3	356.1	79.6	284.4	327.6	428.6		27.8	5.9
BTW 200–400	145.9	215.5	389.5	89.2	259.1	330.5	373.9		30.9	5.2
BTW 400–630	148.9	216.5	390.3	93.0	269.2	335.2	411.6		30.6	5.1
BTW 630–800	152.9	221.0	374.4	92.6	270.1	335.8	413.1		29.0	4.6
GTW < 50	122.7	218.7	376.7	72.3	279.4	341.2	416.2	619.2	25.4	5.2
GTW 50–100	149.4	220.5	384.7	80.5	278.1	340.2	415.9	599.8	28.3	4.9
GTW 100–200	155.5	222.3	397.1	86.6	278.2	340.1	418.3	579.9	30.4	5.0
GTW 200–400	153.8	222.4	400.9	88.7	282.9	340.9	423.2	519.0	30.8	4.6
GTW 400–630	155.5	224.8	404.8	92.6	286.1	341.9	426.0	496.6	31.2	4.8
GTW 630–800	155.0	222.3	407.3	93.8	272.7	336.3	410.9		31.5	4.7

**Table 3:** Oxidation induction time (OIT) for LDPE and composites.

Particle Size [µm]	Oxidation Induction Time, OIT [min]		
	LDPE SEB 853	1% BTW	1% GTW
<50	0.5	3.5	2.2
50–100		3.4	6.4
100–200		5	7.3
200–400		0.3	1.4
400–630		2.2	0.8
630–800		0.4	1.6

**Table 4:** Thermogravimetric parameters for polyethylene and its composites.

Sample Name	T_95_ [°C]	T_90_ [°C]	T_50_ [°C]	DTG [°C]	Residual Mass [%]
LDPE	434.6	447.5	475.5	479.8	−0.34
BTW < 50	435.5	447.6	474.9	480.8	0.08
BTW 50–100	437.7	450.1	477.1	482.8	0.08
BTW 100–200	435.7	448	475.7	418.2	0.08
BTW 200–400	437.1	449.8	477	483.2	0.05
BTW 400–630	434.3	447.2	475.5	481.4	0.11
BTW 630–800	429.2	445.1	474.7	477.5	0.54
GTW < 50	437.7	449.8	476.5	479.6	0.1
GTW 50–100	438.2	449.6	476.4	482.3	0.1
GTW 100–200	437.1	449.4	476.9	482.6	0.09
GTW 200–400	435.6	448.7	476.4	482.8	0.08
GTW 400–630	434.8	448.8	477.3	483.2	0.26
GTW 630–800	434.4	447.2	475.5	482.4	0.06

## Data Availability

The data that support the findings of this study are available from the corresponding author upon reasonable request.
